# Evaluation of Fasting Glucose-Insulin-C-Peptide-Derived Metabolic Indices for Identifying Metabolic Syndrome in Young, Healthy Adults

**DOI:** 10.3390/nu16132135

**Published:** 2024-07-04

**Authors:** Irina Bianca Kosovski, Dana Ghiga, Cristina Nicoleta Ciurea, Dragos Constantin Cucoranu, Liliana Demian, Florina Ioana Gliga, Anca Bacârea

**Affiliations:** 1 Department of Pathophysiology, George Emil Palade University of Medicine, Pharmacy, Science and Technology of Târgu Mureș, 540139 Târgu Mureș, Romania; bianca.kosovski@umfst.ro (I.B.K.); florina.gliga@umfst.ro (F.I.G.); anca.bacarea@umfst.ro (A.B.); 2 Doctoral School, George Emil Palade University of Medicine, Pharmacy, Science and Technology of Târgu Mureș, 540139 Târgu Mureș, Romania; 3 Department of Research Methodology, George Emil Palade University of Medicine, Pharmacy, Science and Technology of Târgu Mureș, 540139 Târgu Mureș, Romania; 4 Department of Microbiology, George Emil Palade University of Medicine, Pharmacy, Science and Technology of Târgu Mureș, 540139 Târgu Mureș, Romania; cristina.ciurea@umfst.ro; 5 Department of Radiology, Mures County Emergency Hospital, 540136 Târgu Mureș, Romania; cucoranud@gmail.com; 6 Center for Advanced Medical and Pharmaceutical Research, George Emil Palade University of Medicine, Pharmacy, Sciences and Technology Târgu Mureș, 540139 Târgu Mureș, Romania; lilidemian@yahoo.com

**Keywords:** metabolic syndrome, insulin resistance, inflammation, young healthy adults, HOMA-IR, HOMA-BETA, disposition index, 20/C-peptide*glucose, QUICKI, C-peptide index

## Abstract

Metabolic syndrome (MetS) is a condition defined by a cluster of symptoms, including excessive adipose tissue, impaired glucose homeostasis, dyslipidemia, and high blood pressure (BP). We aimed to evaluate the correlation between the MetS criteria (IDF) and fasting glucose-insulin-C-peptide-derived indices in a cohort of 128 healthy young adults who were 20–35 years old at the time of this study. We measured fasting serum glucose, insulin, C-peptide (CP), HDL-cholesterol, triglycerides, and hsCRP; HOMA-IR INS, HOMA-IR CP1, HOMA-IR CP2, HOMA-BETA, HOMA-BETA CP, QUICKI, disposition index (DI), CP index (CPI), and 20/C-peptide*glucose. Significant correlations were found between BMI and all HOMA indices, QUICKI, and CPI; waist circumferences and HOMA-IR INS, HOMA-BETA, and QUICKI (for both sexes); glucose and HOMA-IR INS/CP1/CP2, HOMA-BETA CP, DI, and QUICKI; HDL-cholesterol and HOMA-IR INS, HOMA-BETA, and QUICKI for males and females only with QUICKI; triglycerides and HOMA-IR INS, HOMA-BETA, and QUICKI; systolic BP and HOMA-IR INS, HOMA-BETA; diastolic BP and DI. The cut-off values for HOMA-IR INS, HOMA-BETA, and QUICKI in the combined group (females + males) were 1.855, 82.250, 0.355; 2.115, 106.370, 0.345 for males; 1.805, 71.305, 0.355 for females. A stronger correlation was found between males’ indices and hsCRP. In conclusion, CP-derived indices do not add significant information, and the male sex is more predisposed to MetS.

## 1. Introduction

Metabolic syndrome (MetS) is defined as a cluster of conditions typically characterized by a gradual progression that significantly increases the risk of cardiovascular diseases (CVD) and type 2 diabetes mellitus (T2DM) above the sum of the risks associated with each abnormality [1].

Despite the varied classifications proposed by several prominent international organizations and expert groups aiming to refine the definition of MetS, all converged to adopt the same traditional criteria: excessive adipose tissue (AT), impaired glucose homeostasis, dyslipidemia (elevated triglyceride levels and decreased HDL-cholesterol) and high blood pressure (BP). The primary differences among the proposed classifications lie in their perspectives; for example, the World Health Organization (WHO, 1998) adopts a glucocentric approach, the International Diabetes Federation (IDF, 2005) prioritizes obesity, while the Adult Treatment Panel III of the National Cholesterol Education Program (NCEP-ATP III, 2001) focuses on CVD prediction [2,3,4]. For this study, we opted to utilize the classification proposed by IDF due to its inclusion of additional factors beyond traditional criteria, such as ethnicity and sex-specific waist circumference (WC) thresholds, thus enhancing the comprehensiveness of the classification in the MetS context [3].

In the context of globalization, alongside economic and political development, the incidence of diseases continues to escalate, driven by the combination of sedentarism, overeating, and poor nutrition. The COVID-19 pandemic, through its imposed restrictions, exacerbates the incidence of obesity, further fueling the development of MetS. The WHO reported that in 2022, 1 in 8 people in the world were obese; respectively, 43% of adults were overweight and 16% were obese [5]. Currently, the prevalence of MetS is on the rise worldwide. A meta-analysis study, including 28 million individuals, reported that the prevalence of MetS in the European Region, based on the IDF criteria, is 31.4%. Furthermore, the study found that globally, the prevalence of MetS tends to increase with the level of income in a country [6].

The pathogenesis of MetS has been suggested to be central obesity and insulin resistance (IR). The hyperinsulinemic-euglycemic clamp remains the gold standard for identifying IR; however, due to its technical complexity, simpler indirect methods have been utilized for epidemiological and clinical studies. These methods involve the advantage of using a serum sample assayed for glucose, C-peptide (CP), and insulin, either in a fasting state or after stimulation, followed by the calculation of various indices, including the Homeostasis Model Assessment of Insulin Resistance (HOMA-IR INS) and its CP derived indices (HOMA-IR CP1; HOMA-IR CP2); the Homeostasis Model Assessment of β-cell function (HOMA-BETA) and its derived HOMA-BETA CP; the Quantitative Insulin Sensitivity Check Index (QUICKI); the disposition index (DI); the C-peptide index (CPI), and the 20/C-peptide*glucose index [7,8,9,10,11,12,13].

Over recent decades, the identification and understanding of disturbances associated with MetS have been crucial for early detection, prevention, and management of related health complications. In the context of apparently healthy individuals with normal weight, overweight, and obesity, we have chosen to investigate MetS in a group of young adults aged 20 to 35 years old. It has been reported that from the early age of 22, glucose levels are influenced by WC and body mass index (BMI), which are the main diagnostic criteria for MetS according to the International Diabetes Federation’s (IDF) classification [14]. Furthermore, risky behaviors that promote obesity are widespread, even present during adolescence in Mures County [15,16]. The novelty of the study lies in the use of non-obese participants as a control group, along with the apparently healthy status of the study group. These criteria help exclude the secondary effects of other comorbidities from the results, providing a foundation for the public health sector to integrate personalized prevention strategies. On the other hand, studies like ours support worldwide health data collection, raising awareness and serving as indicators for the dimensions of MetS.

This study aims to achieve three objectives: (1) to evaluate the prevalence of MetS and the correlations of several fasting glucose-insulin-C-peptide derived metabolic indices (including HOMA-IR INS, HOMA-IR CP1, HOMA-IR CP2, HOMA-BETA, HOMA-BETA CP, QUICKI, DI, CPI, and 20/C-peptide*glucose) with MetS criteria among young, healthy adults aged 20 to 35 years old; (2) to assess subclinical inflammatory syndrome by exploring the correlation between high-sensitivity C-reactive protein (hsCRP) and the metabolic indices; (3) to determine the cut-off values for commonly used metabolic indices (HOMA-IR, INS, HOMA-BETA, QUICKI) to identify MetS.

## 2. Materials and Methods

### 2.1. The Study Protocol and Population

This cross-sectional study was approved by the Ethics Committee of the County Clinical Emergency Hospital of Târgu Mureș (Decision no. Ad.29270/08.12.2020), and the protocol aligns with the principles outlined in the Declaration of Helsinki. The participants were recruited voluntarily following the dissemination of information about the study across multiple public social platforms and groups, thereby engaging individuals who met the inclusion criteria. After obtaining informed consent from all participants, a questionnaire was applied to assess lifestyle factors and medical history, followed by anthropometric measurements and blood collection.

The inclusion criteria comprise individuals in an apparently clinically healthy state within the age range of 20–35 years. A healthy status was characterized by individuals without recent acute episodes (≥30 days have passed since the last acute episode) or chronic pathologies, not undergoing drug treatment (anti-inflammatory, antihypertensive, anti glycemic, antilipidemic, etc.), and not in the pregnant or postpartum stage for women.

### 2.2. Laboratory Methods

#### 2.2.1. Blood Sample Collection

Venous blood was obtained in the morning after an overnight fast, using BD Vacutainers^®^ (Becton, Dickinson and Company, Franklin Lakes, NJ, USA) equipped with a clot activator agent and gel serum separator. Following centrifugation, all serum samples were aliquoted into Eppendorf^®^ tubes (Eppendorf, Hamburg, Germany) and stored at −80 degrees Celsius until the entire study group was recruited and tests were performed.

#### 2.2.2. Turbidimetric Assay

The inflammatory marker hsCRP was assessed using the turbidimetric assay with the COBAS INTEGRA CRP HS kit (Cat. No. 04628918190, ROCHE), and the results were quantified with the COBAS INTEGRA 400 plus biochemistry analyzer (ROCHE, F. Hoffmann–La Roche, Ltd., Basel, Switzerland), and expressed in mg/L.

#### 2.2.3. Colorimetric Assay

All colorimetric assays were conducted on serum samples using the COBAS INTEGRA 400 plus chemistry analyzer (ROCHE, F. Hoffmann–La Roche, Ltd., Basel, Switzerland) with specific methods and test principles for each analyte.

Triglyceride concentrations were evaluated with TRIGL reagent (REF. 20767107322, ROCHE, F. Hoffmann–La Roche, Ltd., Basel, Switzerland). The enzymatic colorimetric method utilizing lipoprotein lipase was used, and the results were expressed in mg/dL.

High-density lipoprotein cholesterol (HDL-cholesterol) serum concentrations were quantified with the HDLC4 reagent (REF. 07528566190, ROCHE, F. Hoffmann–La Roche, Ltd., Basel, Switzerland). A homogeneous enzymatic colorimetric assay was applied, utilizing PEG-cholesterol esterase. The results were expressed in mg/dL.

Glucose concentration was quantified using the GLUC3 reagent (REF. 04404483190, ROCHE, F. Hoffmann–La Roche, Ltd., Basel, Switzerland) based on the enzymatic reference method with hexokinase. The results were expressed in mg/dL. The conversion factor for glucose was applied as specified in the reagent insert mmol/L = mg/dL/18.02.

#### 2.2.4. Chemiluminescent Immunoassay

The serum concentrations of insulin and CP were assessed using the IMMULITE^®^ 2000 Insulin reagent (Cat. No. L2KIN2, DPC^®^, Los Angeles, CA, USA) and the IMMULITE^®^ 2000 C-Peptide reagent (Cat. No. L2KPE2, DPC^®^, Los Angeles, CA, USA) with the IMMULITE^®^ 2000 XPi Immunoassay System (Siemens Healthcare GmbH, Erlangen, Germany). The insulin assay utilized a solid-phase and two-site chemiluminescent enzyme immunoassay method, while the CP assay applied a competitive chemiluminescent enzyme immunoassay method. The results were quantified and expressed in µIU/mL for insulin and ng/mL for CP.

### 2.3. Anthropometric Measurements

The weight (kg) and BMI were determined using the TANITA BC-1000 Inner Scan^®^ scale (TANITA Corporation, Tokyo, Japan). BMI was calculated according to the WHO formula: BMI = weight/height^2^ (kg/m^2^) [5]. Additionally, WC (cm) and height (cm) were measured using a centimeter and a stadiometer, respectively.

Blood pressure was assessed at the level of the dominant hand with an upper arm electronic sphygmomanometer (OMRON 5 Series Wireless Bluetooth^®^, OMRON Healthcare, Inc., Kyoto, Japan).

### 2.4. Definitions, Classifications, and Formulas

MetS was evaluated using the IDF criteria as (1) central obesity defined by ethnicity-specific values (for Europid: WC ≥94 cm for men and ≥80 cm for women; or assumed if BMI is ≥30 kg/m^2^); (2) fasting serum glucose ≥100 mg/dL (≥5.6 mmol/L) or a previous diagnosis of T2DM; (3) fasting serum triglycerides ≥150 mg/dL (≥1.7 mmol/L) or undergoing antilipidemic treatment; (4) fasting serum HDL-cholesterol < 40 mg/dL (<1.03 mmol/L) for men and <50 mg/dL (<1.29 mmol/L) for women; (5) systolic BP (SBP) ≥130 and/or diastolic BP (DBP) ≥ 85 mmHg or undergoing antihypertensive treatment. MetS was defined as the presence of three or more criteria, including criteria (1) plus any two additional criteria from (2) to (5) [3].

The BMI was interpreted according to WHO criteria as follows: normal weight (18.50–24.99), overweight (25.00–29.99), and obese (≥30.00) [5].

The fasting glucose-insulin-C-peptide derived metabolic indices were considered: HOMA-IR INS, HOMA-IR CP1, HOMA-IR CP2, HOMA-BETA, HOMA-BETA CP, QUICKI, DI, CPI, and 20/C-peptide*glucose [7,8,9,10,11,12,13]. The metabolic indices were evaluated using the following formulas:

$$HOMA\text{-}IR\;INS=\frac{fasting\;glucose\;(mmol/L)\times fasting\;insulin\;(µU/mL)}{22.5}$$

$$HOMA\text{-}IR\;CP1=\frac{fasting\;glucose\;(mmol/L)\times fasting\;C\text{-}peptide\;(ng/mL)}{22.5}$$

$$HOMA\text{-}IR\;CP2=1.5+\frac{fasting\;glucose\;(mg/dL)\times fasting\;C\text{-}peptide\;(ng/mL)}{2800}$$

$$HOMA\text{-}BETA=\frac{fasting\;insulin\;(µIU/mL)\times 20}{fasting\;glucose\;(mmol/L)-3.5}$$

$$HOMA\text{-}BETA\;CP=\frac{fasting\;C\text{-}peptide\;(ng/mL)\times 20}{fastingglucose\;(mmol/L)-3.5}$$

$$DI=\frac{HOMA\text{-}BETA}{HOMA\text{-}IRINS}$$

$$20/C\text{-}peptide*glucose=\frac{20}{fasting\;C\text{-}peptide\;(ng/mL)\times fasting\;glucose\;(mg/dL)}$$

$$QUICKI=\frac{1}{log(fasting\;insulin,\;µIU/mL)+log(fasting\;glucose,\;mg/dL)}$$

$$CPI=\frac{fasting\;C\text{-}peptide\;(ng/mL)}{fasting\;glucose\;(mmol/L)}\times 100$$

### 2.5. Statistical Methods

The statistical analysis included both descriptive (since the mean, standard deviation, median, frequency, and percentages were assessed) and inferential statistics. The *t*-student test, a parametric test for comparing means, and the Mann-Whitney test, a non-parametric test for comparing medians, were used. The Pearson test assessed the correlation (i.e., the measure of the association between the quantitative variables). The Chi-squared test and Fisher’s test were conducted to evaluate the association between qualitative variables. To evaluate the accuracy, the Area Under the ROC Curve (AUC) was calculated, and to assess the diagnostic test’s performance over the range of possible cut points for the predictor variable was performed a ROC curve. A significance threshold of *p* < 0.05 was chosen. The statistical analysis was conducted using the demo version of the GraphPad Prism program.

## 3. Results

### 3.1. General Characteristics of the Cohort

A cohort of 128 subjects selected from 167 participants met the eligibility criteria. They were divided into two groups according to IDF MetS criteria: MetS(+) (30, 23.44%) and MetS(−) (98, 76.56%). The general cohort description, including risk behaviors (defined as alcohol and tobacco consumption) as well as personal and family history of excessive AT, is presented in Table 1. The median age of the subjects MetS(+) is higher compared to the group MetS(−), 29 years old vs. 27 years old. In the MetS(+) group, males represent the majority (21, 70%) compared to females (9, 30%), while in the group MetS(−), a reciprocal distribution of sexes can be observed (39, 39.80% males vs 59, 60.20% females).

Furthermore, the presence in the personal history of excessive AT expressed as overweight or obesity, as well as the duration (i.e., the number of years), show a significant difference between groups (*p* = 0.0003; *p* = 0.0034). The group MetS(+) exhibited a greater occurrence of excessive AT in their family history (80.00% vs. 61.22%). However, this association with MetS was not significant (*p* = 0.0785). Risk behaviors such as tobacco and alcohol consumption did not indicate a significant association between consumer status and the presence of MetS (*p* = 0.8082; *p* = 0.3356).

### 3.2. Analysis of the Anthropometric Measurements, Blood Parameters, and Metabolic Indices

A detailed description of the anthropometric measurements, blood parameters, and metabolic indices can be found in Table 2. It was observed that in the group MetS(+), both height and weight, as well as BMI, presented higher mean/median values compared to the group MetS(−) (176.60 vs. 171.20; 101.80 vs. 76.70; 33.75 vs. 26.60), with significant differences (*p* = 0.0146; *p* < 0.0001; *p* < 0.0001). Additionally, the mean values of the WC were significantly higher in the subjects MetS(+) (108.50 vs. 86.06, *p* < 0.0001).

Furthermore, both fractions of BP, SBP, and DBP showed significantly higher median values in the MetS(+) group (127.00 vs. 115.00 with *p* < 0.0001; 81.00 vs. 76.50 with *p* = 0.0002, respectively).

The glucose metabolism was investigated by measuring fasting serum glucose, fasting serum insulin, and fasting serum CP. All three markers showed increased mean or median concentrations in the MetS(+) group, but only insulin was significant (glucose: *p* = 0.4390; insulin: *p* = 0.0005; CP: *p* = 0.5836). On the other hand, lipid metabolism revealed significant differences between the studied groups (*p* < 0.0001) across the investigated parameters: a reduction in HDL-cholesterol levels (38.44 vs. 52.46) and an elevation in triglyceride levels (156.40 vs. 95.96) were found in the MetS(+) group. In addition, a significant variation was observed in hsCRP concentration, with subjects with MetS(+) demonstrating higher levels compared to the group MetS(−), 1.96 vs. 0.79 (*p* = 0.0002).

As presented in Table 2, when comparing the median values of all subtypes of the HOMA indices (INS, CP1, CP2, BETA, BETA CP), only HOMA-IR INS and HOMA-BETA showed significant differences (*p* = 0.0008; *p* = 0.0005). Except for HOMA-IR CP1 (0.17 vs. 0.21), HOMA-IR CP2 (1.52 vs. 1.53), and HOMA-BETA CP (12.96 vs. 13.69), the median values of all HOMA index subtypes were elevated in the MetS(+) group.

QUICKI demonstrated a significantly decreased mean value in the group MetS(+) (0.33 vs. 0.37, *p* = 0.0005). The median values for DI and 20/C-peptide*glucose were higher in the MetS(+) group (60.79 vs. 59.22; 0.28 vs. 0.23) but without statistical significance (*p* = 0.6346; *p* = 0.7272). On the other hand, in the MetS(+) group, the median value of the CPI demonstrated a reduction (17.97 vs. 20.94; *p* = 0.5931).

### 3.3. The Analysis of Correlations between MetS Components and Metabolic Indices

All the correlations between MetS components and the indices are detailed in Table 3 and Table 4. For a comprehensive analysis, the correlations for each MetS component will be treated collectively within the same paragraph based on the values extracted from both Table 3 and Table 4.

With the exception of DI and 20/C-peptide*glucose (*p* = 0.8829; *p* = 0.2616), BMI correlated significantly with the other metabolic indices (*p* < 0.05). Positive correlations were found between BMI and HOMA-IR INS (r = 0.5136), HOMA-IR CP1 (r = 0.2361), HOMA-IR CP2 (r = 0.2428), HOMA-BETA (r = 0.5090), HOMA-BETA CP (r = 0.1737), DI (r = 0.0131) and CPI (r = 0.2200). Alternatively, BMI correlated negatively with QUICKI (r = −0.4695) and 20/C-peptide*glucose (r = −0.0999).

The analysis of WC correlations was stratified by sex according to specific cut-offs. Among females, except for DI (r = −0.0096), QUICKI (r = −0.2761), and 20/C-peptide*glucose (r = −0.0363), all indices showed a positive correlation with WC. Significant correlations were observed between WC and HOMA-IR INS (*p* = 0.0082), HOMA-BETA (*p* = 0.0482), and QUICKI (*p* = 0.0227). In addition, among males, positive correlations were observed with all indices except for QUICKI (r = −0.5877) and 20/C-peptide*glucose (r = −0.0135). Similar to females, the significant correlations with the indices were maintained: HOMA-IR INS (*p* < 0.0001), HOMA-BETA (*p* < 0.0001), and QUICKI (*p* < 0.0001), respectively.

Furthermore, glucose concentration shows significant correlations with all the indices (*p* < 0.05), except for HOMA-BETA (*p* = 0.1191), 20/C-peptide*glucose (*p* = 0.0906), and the CPI (*p* = 0.2738). An increase in glucose level correlates with higher values of HOMA-IR INS (r = 0.3676), HOMA-IR CP1 (r = 0.3129), HOMA-IR CP2 (r = 0.3099), and CPI (r = 0.0974). However, HOMA-BETA (r = −0.1384), HOMA-BETA CP (r = −0.2691), DI (r = −0.8432), QUICKI (r = −0.3603), and 20/C-peptide*glucose (r = −0.1502) display negative correlations with glycemia.

The correlations of HDL-cholesterol were divided by gender based on MetS cut-off values. Among females, a positive correlation was observed between HDL-cholesterol and DI, QUICKI, and 20/C-peptide*glucose, with only QUICKI showing a significant correlation (*p* = 0.0416, respectively). All other indices showed nonsignificant negative correlations with HDL-cholesterol concentration (*p* > 0.05). Conversely, among males, a negative correlation was noted between all indices and HDL-cholesterol, except for DI and QUICKI (r = 0.0452; r = 0.3251). Significant correlations were evident between HDL-cholesterol and HOMA-IR INS (r = −0.3684; *p* = 0.0038), HOMA-BETA (r = −0.4502; *p* = 0.0003), and QUICKI (r = 0.3251; *p* = 0.0113).

Furthermore, except for DI (r = −0.0506), QUICKI (r = −0.3615), and 20/C-peptide*glucose (r = −0.0684), triglycerides were positively correlated with the other indices. Significant correlations were found between triglycerides and HOMA-IR INS (r = 0.2609; *p* = 0.0029), HOMA-BETA (r = 0.2537; *p* = 0.0039), and QUICKI (r = −0.3615; *p* < 0.0001).

SBP noted a negative correlation with QUICKI (r = −0.0953) and 20/C-peptide*glucose (r = −0.0230) and a positive correlation with all other indices. Significant correlations were observed for HOMA-IR INS (*p* = 0.0312) and HOMA-BETA (*p* = 0.0073), both positively associated (r = 0.1906; r = 0.2360). On the other hand, DBP negatively correlated with HOMA-IR CP1 (r = −0.0057), HOMA-IR CP2 (r = −0.0035), QUICKI (r = −0.0529), and 20/C-peptide*glucose (r = −0.0478), all nonsignificant (*p* > 0.05). The only significant correlation observed was between DBP and DI (*p* = 0.0348), which was positive (r = 0.1868).

### 3.4. The Cut-Off Value of HOMA-IR INS, HOMA-BETA, and QUICKI That Defines MetS

**Figure 1:**
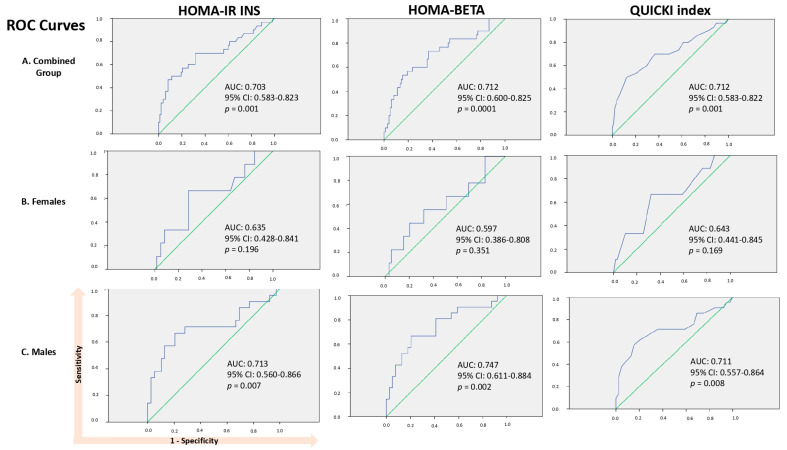
Receiver operating characteristic (ROC) curve for HOMA-IR INS, HOMA-BETA, and QUICKI to determine MetS. HOMA-IR INS: the homeostasis model assessment-estimated insulin resistance, insulin derived index; HOMA-BETA: the homeostasis model assessment of β-cell function; QUICKI: the quantitative insulin sensitivity check index; AUC: Area Under the Curve; 95% CI: confidence interval of 95%.

Based on the significant differences (*p* < 0.05) observed among the study groups in terms of HOMA-IR INS, HOMA-BETA, and QUICKI presented in Table 2, we proceeded to evaluate their diagnostic accuracy through receiver operating characteristic (ROC) curve analysis and calculation of the AUC, separately in the combined group (females + males), females, and males, as illustrated in Figure 1. In the combined group, the statistically significant *p*-values (*p* < 0.05) and AUC suggest that HOMA-IR INS (AUC: 0.703; 95% CI: 0.583–0.823, *p* = 0.001), HOMA-BETA (AUC: 0.712; 95% CI: 0.600–0.825; *p* = 0.0001), and QUICKI (AUC: 0.712; 95% CI: 0.583–0.822; *p* = 0.001) possess discriminatory power in distinguishing between subjects with and without MetS, indicating moderate diagnostic accuracy. Furthermore, when the groups were stratified by sex, the AUC tended to decrease in the female group and increase in the male group (HOMA-IR INS AUC of 0.703 for the combined group vs. 0.635 for females and 0.713 for males; HOMA-BETA AUC of 0.712 for the combined group vs. 0.597 for females and 0.747 for males), except for the QUICKI index (AUC of 0.712 for the combined group vs. 0.643 for females and 0.711 for males). Interestingly, statistical significance was observed exclusively in the male group regarding the accuracy of the test and its ability to differentiate between subjects with and without MetS (*p* = 0.007 for HOMA-IR INS, *p* = 0.002 for HOMA-BETA, and *p* = 0.008 for QUICKI index).

Furthermore, three cut-off values for each index were calculated based on sensitivity (defined as the test’s ability to identify individuals with the disease), specificity (defined as the test’s ability to identify individuals without the disease), and false positive rate (which was set at 1—specificity and was the marker of the probability that a healthy individual will test positive). These values are detailed separately in Table 5 for the combined group (containing both females and males), the female group, and the male group. For HOMA-IR INS, the appropriate cut-off for the combined group, determined based on achieving higher sensitivity and specificity and minimizing false positive rates, was found to be 1.855 (sensitivity: 70.0%; specificity: 68.4%; false positive rate: 31.6%). Compared to the combined group, the optimal HOMA-IR INS cut-off was lower for females, 1.805 (sensitivity: 66.7%, specificity: 71.2%, false positive rate: 28.8%), and higher for males, 2.115 (sensitivity: 71.4%, specificity: 71.8%, false positive rate: 28.2%).

Additionally, for the combined group, the cut-off value for HOMA-BETA was 82.250 (sensitivity: 76.7%, specificity: 54.1%, false positive rate: 45.9%). When analyzed by sex, the optimal cut-off was lower for females at 71.305 (sensitivity: 66.7%, specificity: 49.3%, false positive rate: 50.8%) and higher for males at 106.370 (sensitivity: 81.0%, specificity: 59.0%, false positive rate: 41.0%), maintaining the same trend observed for HOMA-IR INS.

Interestingly, the optimal cut-off values for QUICKI were similar across all three categories: 0.355 (sensitivity: 70.0%, specificity: 63.3%, false positive rate: 36.7%) for the combined group; 0.355 (sensitivity: 66.7%, specificity: 67.8%, false positive rate: 32.2%) for females; 0.345 (sensitivity: 71.4%, specificity: 64.1%, false positive rate: 35.9%) for males.

### 3.5. The Analysis of Low-Grade Inflammatory Syndrome in the MetS Context

Considering the subclinical inflammatory syndrome associated with excessive AT, we aimed to evaluate it within the clinical context of healthy young adults. Therefore, we calculated the correlation of hsCRP with all metabolic indices, as presented in Table 6. In the combined group, hsCRP correlated positively with all indices except for QUICKI and 20/C-peptide*glucose. Additionally, except for DI (*p* = 0.4457), all positive correlations were statistically significant (*p* < 0.05). Furthermore, when stratified by sex, the male group showed significant correlations with all indices except DI (*p* = 0.0554) and 20/C-peptide*glucose (*p* = 0.2174). Apart from QUICKI and 20/C-peptide*glucose, in the male group, all correlations were positive between metabolic indices and hsCRP. The female cohort only exhibited a significant positive correlation with HOMA-IR CP2 (*p* = 0.0415). After analyzing the correlation pattern, it appears that the male group predominantly influences the overall findings. It was observed a negligible discrepancy between the hsCRP of the combined group’s correlation vs. females in HOMA-BETA (r = 0.1808, *p* = 0.0411 vs. r = −0.0229, *p* = 0.8525) and DI (r = 0.0679, *p* = 0.4457 vs. r = −0.0401, *p* = 0.7450), respectively.

## 4. Discussion

A 15% prevalence of MetS was reported among nondiabetic adult Europeans, with an elevated risk of death from all causes, including CVD [17]. Given the variability in classification systems for identifying MetS, our objective was to employ studies utilizing the IDF criteria for defining MetS whenever feasible, as undertaken in this study. This approach was chosen to ensure consistency and facilitate a more comprehensive interpretation of the results.

In our study, we found a higher prevalence of MetS in men compared to females (70.00% vs. 30.00%). Similar to our findings, a study involving Chinese subjects aged 19–44 years old reported a higher prevalence of MetS in men compared to women (21.81% vs. 5.62%), based on the IDF criteria [18]. When the males were divided according to the number of MetS components, it was observed that the higher the number of components, the higher the associated value of HOMA-IR INS, with a value of 2.64 for those meeting ≥3 criteria. A significant positive correlation (*p* < 0.05) was found between HOMA-IR INS and triglycerides, glucose, SBP, DBP, and WC (r = 0.460, r = 0.464, r = 0.362, r = 0.346, r = 0.586). A negative correlation was reported between HOMA-IR INS and HDL-cholesterol (r = −0.357, *p* < 0.05). We observed the same direction of correlation between HOMA-IR INS and the component of the MetS. Furthermore, they emphasized that for males, WC has the strongest correlation with HOMA-IR INS among all components of MetS. Similar to our findings, the highest correlation coefficient (r = 0.6529, *p* < 0.001) was observed between WC and HOMA-IR INS for males (r = 0.6529, *p* < 0.001), followed by BMI (r = 0.5136, *p* < 0.001), thereby supporting the role of AT in IR [18]. Moreover, consistent with our findings, a study conducted in the same demographic area as our study reported a positive and significant correlation between HOMA-IR and all IDF MetS criteria, except for HDL-cholesterol, which exhibited a significant negative association (r = −0.31; *p* = 0.01) [19].

Another study, which involved police officers with a mean age of 41 years and utilized IDF criteria to define MetS, identified a similar increased mean value of HOMA-IR INS in the MetS(+) group (3.05 ± 1.30 vs. 1.89 ± 0.08; *p* < 0.000), consistent with our findings (3.82 ± 3.61 vs. 1.66 ± 1.33, *p* = 0.0008). In line with our results, HOMA-IR INS correlated with all MetS components (*p* < 0.0001), and the calculated cut-off point for MetS diagnosis was 2.66, for which the prevalence for MetS was 38.30% [20]. In our study, a cut-off value of 1.85 showed superior sensitivity, specificity, and false positive rate. A study focusing on Turkish adolescents aged 10–16 years identified a cut-off value for HOMA-IR > 2.52 (with a sensitivity of 83.2% and specificity of 40.4%) to assess the risk of MetS based on IDF criteria [21]. A recent study indicated that elevated HOMA-IR INS values were associated with a significant risk of 1.87 for developing T2DM, 1.46 for non-fatal major adverse cardiovascular events, and 1.35 for hypertension [22].

Moreover, a study involving subjects aged 50 years old found a significant increase in the values of HOMA-IR INS and HOMA-BETA in the MetS(+) group (2.72 vs. 1.37, 97.48 vs. 89.82, *p* < 0.001), as we found it in our study [23]. HOMA-BETA demonstrated a positive correlation with all the MetS criteria, except HDL-cholesterol, with statistical significance observed for WC, BMI, DBP, and HDL-cholesterol (*p* < 0.05). In contrast, the same study revealed significant correlations between all MetS criteria and HOMA-IR INS. Notably, the strongest correlation was observed between HOMA-IR INS, HOMA-BETA, and WC (r = 0.57; r = 0.39; *p* < 0.001). In our study, HOMA-BETA also exhibited a higher correlation with WC (r = 0.5090, *p* < 0.001), similar to HOMA-IR INS. Through regression analysis, they determined that for every 1-point increase in WC, HOMA-IR and HOMA-BETA increased by a mean of 0.1 and 1.97, respectively (*p* < 0.001; *p* = 0.032) [23].

In a study involving non-diabetic Japanese males, a significant positive correlation was observed between QUICKI and BMI (r = 0.434; *p* < 0.001), SBP (r = 0.329; *p* < 0.001), body fat content (%) (r = 0.440; *p* < 0.001), triglycerides (r = 0.246; *p* = 0.006), and HDL-cholesterol (r = 0.194; *p* = 0.029) [24]. Conversely, in our study, a significant negative correlation was found between QUICKI and BMI, WC (for both sexes), and triglycerides, along with a significant positive correlation with HDL cholesterol (for both sexes). Additionally, when comparing HOMA-IR (correlations were found with BMI as r = 0.365 and *p* < 0.001; triglycerides as r = 0.282 and *p* = 0.001) and QUICKI in the non-diabetic Japanese male population, QUICKI demonstrated a stronger association with the criteria for MetS [24].

Corroborating the previously presented data regarding indices such as HOMA-IR INS, HOMA-BETA, and QUICKI, commonly used in MetS, our study calculated optimal thresholds for early detection of this pathology within the specific cohort of healthy young adults. As consequence, we identified that the optimal thresholds of HOMA-IR INS for MetS were 1.855 (sensitivity: 70.0%; specificity: 68.4%; false positive rate: 31.6%): 21 (70.0%) out of 30 subjects MetS(+) presented HOMA-IR INS ≥ 1.855, while 67 (68.4%) out of 98 subjects MetS(−) had HOMA-IR INS < 1.855. A cut-off of 82.250 (sensitivity: 76.7%; specificity: 54.1%; false positive rate: 45.9%) for HOMA-BETA was associated with early MetS detection: 23 (76.7%) out of 30 subjects MetS(+) had HOMA-BETA ≥ 82.250, while 53 (54.1%) out of 98 subjects MetS(−) had HOMA-BETA < 82.250. On the other hand, a value lower than 0.355 (sensitivity: 70.0%; specificity: 63.3%; false positive rate: 36.7%) for QUICKI correlates with MetS detection: 21 (70.0%) out of 30 subjects MetS(+) showed QUICKI ≤ 0.355, while 62 (63.3%) out of 98 subjects MetS(−) had QUICKI > 0.355. More research is needed to determine the value of these metabolic indices in the diagnosis of MetS, but based on these cut-off values, given that none of them meet the criteria for good diagnostic power, they might be used within the context of a diagnostic strategy scheme that involves employing tests with the highest sensitivity in the initial stage, followed by tests with the highest specificity to achieve the most rapid and accurate identification of MetS.

More recently, the concept of utilizing CP instead of insulin in metabolic indices formulas has been introduced, owing to its equimolar secretion with insulin and longer half-life [25]. However, despite the theoretical interchangeability of CP and insulin, the distinctions between CP-based and insulin-based HOMA-IR and HOMA-BETA have not been fully elucidated. Furthermore, in our study, we observed a significantly higher insulin concentration in the MetS(+) group compared to the group MetS(−) (16.41 ± 14.35 vs. 7.49 ± 5.84, *p* = 0.0005), a difference that was not observed at the level of CP (1.57 ± 1.91 vs. 1.12 ± 1.05, *p* = 0.5836). Similar statistical trends were identified when comparing CP-based and insulin-based HOMA-IR and HOMA-BETA indices between the group MetS(+) and the group MetS(−): significant differences were found for insulin-based HOMA indices (HOMA-IR INS: 2.71 vs. 1.33, *p* = 0.0008; HOMA-BETA: 197.70 vs. 79.34, *p* = 0.0005), while non-significant differences were noted for CP HOMA indices (HOMA-IR CP1: 0.17 vs. 0.21, *p* = 0.6552; HOMA-IR CP2: 1.52 vs. 1.53, *p* = 0.7071; HOMA-BETA CP: 12.96 vs. 13.69, *p* = 0.6813). Additionally, a study involving a nondiabetic population with a mean age of 37 years did not identify a significant correlation between HOMA-IR INS and HOMA-IR CP1, nor between HOMA-IR INS and HOMA-IR CP2 (r = 0.043, *p* = 0.697), respectively [26]. The disparities observed can be explained by the distinct metabolic clearance rates of CP and insulin, along with IR in T2DM associated with MetS, impacting their accumulation. Therefore, a prodromal hyperinsulinemic phase often precedes the progressive decline in beta-cell function, which eventually modifies CP concentration [27]. By comparing a nondiabetic group to a diabetic one (T2DM), a study found similar nonsignificant differences between CP (861.18 ± 63.66 mmol/L vs. 951.17 ± 100.69 mmol/L) and insulin (16.71 ± 2.02 µU/mL vs. 25.25 ± 5.39 µU/mL). However, when correlations were calculated, both markers showed a significant positive correlation with HOMA-IR in both groups (CP: for the diabetic group r = 0.652 and for the nondiabetic group r = 0.500; insulin: for the diabetic group: r = 0.961 and for the nondiabetic group: r = 0.975; for all the correlation *p* = 0.001) [28]. Overall, after analyzing the presented data, it seems that including CP in HOMA measurements does not add additional value. Furthermore, another disadvantage of CP that does not support its use in HOMA formulas is its higher cost compared to serum insulin determination.

In our study, DI expressed a negative correlation with WC, glycemia, and TG and a positive correlation with BMI, HDL cholesterol, SBP, and DBP. The only significant correlation was found between DI and glycemia (r = −0.8432; *p* < 0.0001) and DBP (r = 0.1868; *p* = 0.0348). In a study comprising non-diabetic individuals aged 18 to 60 years, employing NCEP/ATP III criteria, the DI exhibited an inverse association with the number of MetS components. Furthermore, univariate analysis demonstrated significant negative correlations with age, glucose, WC, SBP, and triglycerides and significant positive correlations with HDL-cholesterol, sustaining our results [29].

While studies investigating the correlation between 20/C-peptide*glucose and CPI with MetS in an apparently healthy young adult cohort have not yet been published, our study serves as a pilot investigation. However, a study involving Japanese patients with T2DM with a mean age of 53.2 ± 12.7 years reported that 20/C-peptide*glucose is a more effective index compared to HOMA-IR in patients with mild IR [13]. In our study, we observed that 20/C-peptide*glucose exhibited a non-significant negative correlation with all MetS diagnostic criteria, except for HDL-cholesterol, which showed a positive correlation (r = 0.1007, *p* = 0.4140).

Nevertheless, both MetS and obesity are associated with subclinical inflammatory syndrome. In our previous study, utilizing the same cohort of young, healthy adults but subclassified based on BMI, we demonstrated the presence of subclinical inflammation associated with excessive AT even in this group of healthy subjects [30]. Therefore, we aimed to investigate the correlation between hsCRP, as a part of the ”platinum standard” criteria for MetS, and the metabolic indices [31]. Apart from DI, QUICKI, and 20/C-peptide*glucose, all the metabolic indices showed a significant positive correlation with hsCRP in the combined group (females + males). Based on the correlation coefficient values (r) and the higher number of significant correlations between hsCRP and the metabolic indices, our study identified a stronger association in the male group compared to the female group. This observation may be attributed to the higher prevalence of males with MetS(+) compared to females (70% males vs. 30% females), consistent with the increased prevalence of MetS in this age group as well. In the study of police officers, it was found that HOMA-IR INS significantly correlated with CRP (r = 0.25, *p* < 0.05) and, additionally, with TNF-α (r = 0.17; *p* < 0.05) [20]. Furthermore, in our study, the MetS(+) group exhibited a significantly higher concentration of hsCRP compared to the MetS(−) group: 3.19 ± 3.85 (1.96) vs. 1.51 ± 2.78 (0.79) (*p* = 0.0002). Consistent with our findings, another study reported similar differences between the MetS(+) and control groups for hsCRP: 3.7 ± 2.5 vs. 2.4 ± 1.3 (*p* < 0.05) [32]. Furthermore, to complete the inflammatory profile, other inflammatory markers were also assessed, revealing significantly higher concentrations in the MetS(+) group compared to the control group: TNF-α was 11.4 ± 7.5 vs. 3.6 ± 3.6; IL-6 was 3.4 ± 3.2 vs. 1.3 ± 1.4; and PAI-1 active was 16.4 ± 10.0 vs. 7.4 ± 4.0 [32].

However, one limitation of our study stems from the relatively small cohort size and the cross-sectional design. The small study cohort and the sex distribution imbalance may restrict the generalizability of our findings to broader populations, as it may not adequately represent the diversity present within the target demographic. Additionally, the cross-sectional nature of the study limits our ability to establish causality or determine the temporal sequence of events. Longitudinal studies with larger sample sizes would be beneficial to validate our findings and provide deeper insights into the relationships observed.

## 5. Conclusions

Drawing from the quotation “prevention is cheaper than treatment”, these data underscore the value of utilizing metabolic indices in epidemiological studies of MetS and emphasize the need for targeted public health interventions within this age group, which is often underestimated in terms of risk for chronic diseases. Based on our cohort analysis, substituting insulin with CP does not offer substantial valuable insights for early detection of MetS. Scientifically, testing multiple indices is pivotal. Nevertheless, when contemplating widespread adoption in public health, it is crucial to assess and incorporate cost-effectiveness into the final decision regarding its use as a diagnostic or screening tool.

In our study, the cut-off values for HOMA-IR INS, HOMA-BETA, and QUICKI for early MetS detection in the combined group (females + males) were 1.855, 82.250, and 0.355; for males were 2.115, 106.370, and 0.345; for females 1.805, 71.305, and 0.355. Further studies involving larger cohorts and diverse ethnic groups are needed to establish specific cut-off values for these indices, which will further facilitate the early identification of individuals at risk for MetS. In the combined group, the inflammatory biomarker hsCRP correlated positively with all indices except for QUICKI and 20/C-peptide*glucose, once again highlighting the active metabolic role of adipose tissue. By comparing all presented dates, the male sex is more affected by MetS. Integrating these findings into public health practices can improve screening protocols, personalized interventions (especially age and sex-directed), and overall management strategies aimed at reducing the burden of MetS and its associated complications.

## Figures and Tables

**Table 1:** The general cohort description includes a history of excessive adipose tissue and risk behaviors.

Criteria		MetS(+) (n = 30)	MetS(−) (n = 98)	*p*-Value
Sex	Male—No (%)	21.00 (70.00)	39.00 (39.80)	0.0060 ⸷
	Female—No(%)	9.00 (30.00)	59.00 (60.20)	
Age (years)—mean ± SD (median)		29.50 ± 4.13 (29.00)	27.62 ± 4.37 (27.00)	0.0369 *
Tabacco user	Yes—No (%)	8.00 (26.67)	23.00 (23.47)	0.8082 ⸷
	No—No (%)	22.00 (73.33)	75.00 (76.53)	
Alcohol	Yes—No (%)	5.00 (16.67)	26.00 (26.53)	0.3356 †
	No—No (%)	25.00 (83.33)	72.00 (73.47)	
Overweight or obese in personal history	Yes—No (%)	26.00 (86.67)	49.00 (50.00)	0.0003 †
	No—No (%)	4.00 (13.33)	49.00 (50.00)	
Years of being overweight/obese in personal history—mean ± SD (median)		13.00 ± 9.20 (10.00)	6.93 ± 5.91 (5.00)	0.0034 *
Overweight or obese in family history	Yes—No (%)	24.00 (80.00)	60.00 (61.22)	0.0785 †
	No—No (%)	6.00 (20.00)	38.00 (38.78)	

MetS: metabolic syndrome; no: number; SD: standard deviation; ⸷ Chi-square test; † Fisher test; * Mann-Whitney test.

**Table 2:** Description of the anthropometric measurements, blood parameters, and metabolic indices.

Criteria	MetS(+) (n = 30) Mean ± SD (Median)	MetS(−) (n = 98) Mean ± SD (Median)	*p*-Value
Anthropometric measurements			
Height (cm)	176.60 ± 10.95 (177.50)	171.20 ± 10.23 (171.00)	0.0146 *
Weight (kg)	108.10 ± 24.81 (101.80)	77.53 ± 19.57 (76.70)	<0.0001 **
Body mass index	34.51 ± 6.53 (33.75)	26.18 ± 4.84 (26.60)	<0.0001 **
Waist circumference (cm)	108.50 ± 14.28 (106.00)	86.06 ± 14.30 (86.50)	<0.0001 *
Systolic blood pressure (mmHg)	126.70 ± 8.10 (127.00)	115.20 ± 9.93 (115.00)	<0.0001 **
Diastolic blood pressure (mmHg)	82.47 ± 6.45 (81.00)	76.45 ± 7.32 (76.50)	0.0002 **
Blood parameters			
Glucose (mg/dL)	90.55 ± 10.10 (89.83)	89.25 ± 7.29 (90.38)	0.4390 *
Insulin (µIU/mL)	16.41 ± 14.35 (12.20)	7.49 ± 5.84 (5.97)	0.0005 **
C-peptide (ng/mL)	1.57 ± 1.91 (0.82)	1.12 ± 1.05 (1.02)	0.5836 **
HDL-cholesterol (mg/dL)	38.44 ± 6.05 (38.16)	52.46 ± 10.95 (51.95)	<0.0001 *
Triglyceride (mg/dL)	156.40 ± 62.69 (154.00)	95.96 ± 30.96 (94.97)	<0.0001 *
hsCRP (mg/L)	3.19 ± 3.85 (1.96)	1.51 ± 2.78 (0.79)	0.0002 **
Metabolic indices			
HOMA-IR INS	3.82 ± 3.61 (2.71)	1.66 ± 1.33 (1.33)	0.0008 **
HOMA-IR CP1	0.37 ± 0.48 (0.17)	0.24 ± 0.23 (0.21)	0.6552 **
HOMA-IR CP2	1.55 ± 0.07 (1.52)	1.53 ± 0.03 (1.53)	0.7071 **
HOMA-BETA	217.70 ± 176.30 (197.70)	110.40 ± 90.59 (79.34)	0.0005 **
HOMA-BETA CP	22.21 ± 28.11 (12.96)	17.40 ± 19.75 (13.69)	0.6813 **
QUICKI	0.33 ± 0.05 (0.32)	0.37 ± 0.04 (0.37)	0.0005 *
Disposition index	75.30 ± 56.16 (60.79)	71.47 ± 34.59 (59.22)	0.6346 **
20/C-peptide*glucose	0.79 ± 0.90 (0.28)	0.78 ± 0.88 (0.23)	0.7272 **
C-peptide index	30.22 ± 34.42 (17.97)	22.82 ± 21.32 (20.94)	0.5931 **

HDL-cholesterol: high-density lipoprotein cholesterol; hsCRP: high-sensitivity C-reactive protein; HOMA-IR INS: the homeostasis model assessment-estimated insulin resistance index, insulin derived index; HOMA-IR CP1: the homeostasis model assessment-estimated insulin resistance, C-peptide 1 derived index; HOMA-IR CP2: the homeostasis model assessment-estimated insulin resistance, C-peptide 2 derived index; HOMA-BETA: the homeostasis model assessment of β-cell function; HOMA-BETA CP: the homeostasis model assessment of β-cell function, CP derived index; QUICKI: the quantitative insulin sensitivity check index; * *t*-Student test; ** Mann-Whitney test.

**Table 3:** Correlations of the MetS components with the metabolic indices: HOMA-IR INS, HOMA-IR CP1, HOMA-IR CP2, HOMA-BETA, and HOMA-BETA CP.

	**HOMA-IR INS**		**HOMA-IR CP1**		**HOMA-IR CP2**		**HOMA-BETA**		**HOMA-BETA CP**	
	**r** **CI (95%)**	***p*** **-Value**	**r** **CI (95%)**	***p*** **-Value**	**r** **CI (95%)**	***p*** **-Value**	**r** **CI (95%)**	***p*** **-Value**	**r** **CI (95%)**	***p*** **-Value**
BMI										
	0.5136 0.3733 to 0.6310	<0.0001	0.2361 0.0652 to 0.3935	0.0073	0.2428 0.0722 to 0.3995	0.0058	0.5090 0.3680 to 0.6272	<0.0001	0.1737 0.0001 to 0.3371	0.0499
Waist circumference (cm)										
Females	0.3179 0.0859 to 0.5172	0.0082	0.0883 −0.1533 to 0.3201	0.4736	0.0975 −0.1443 to 0.3284	0.4287	0.2405 0.0020 to 0.4530	0.0482	0.0615 −0.1796 to 0.2957	0.6181
Males	0.6529 0.4782 to 0.7779	<0.0001	0.2499 −0.0043 to 0.4738	0.0541	0.2493 −0.0049 to 0.4734	0.0547	0.6982 0.5399 to 0.8087	<0.0001	0.1362 −0.1221 to 0.3771	0.2996
Glucose (mg/dL)										
	0.3676 0.2073 to 0.5087	<0.0001	0.3129 0.1474 to 0.4614	0.0003	0.3099 0.1441 to 0.4588	0.0004	−0.1384 −0.3047 to 0.0359	0.1191	−0.2691 −0.4229 to −0.1002	0.0021
HDL-cholesterol (mg/dL)										
Females	−0.1977 −0.4165 to 0.0428	0.1061	−0.1380 −0.3645 to 0.1039	0.2617	−0.1360 −0.3627 to 0.1059	0.2687	−0.1516 −0.3765 to 0.0901	0.2172	−0.0844 −0.3166 to 0.1572	0.4934
Males	−0.3684 −0.5691 to −0.1262	0.0038	−0.1572 −0.3954 to 0.1008	0.2302	−0.1564 −0.3947 to 0.1016	0.2327	−0.4502 −0.6320 to −0.2216	0.0003	−0.1735 −0.4094 to 0.0841	0.1849
Triglyceride (mg/dL)										
	0.2609 0.0914 to 0.4157	0.0029	0.1058 −0.0690 to 0.2743	0.2345	0.1028 −0.0720 to 0.2715	0.2481	0.2537 0.0838 to 0.4093	0.0039	0.0876 −0.0873 to 0.2573	0.3255
Blood pressure (mmHg)										
SBP	0.1906 0.0175 to 0.3525	0.0312	0.0919 −0.0829 to 0.2614	0.3017	0.0934 −0.0814 to 0.2628	0.2940	0.2360 0.0651 to 0.3935	0.0073	0.1143 −0.0604 to 0.2823	0.1988
DBP	0.0887 −0.0861 to 0.2584	0.3191	−0.0057 −0.1792 to 0.1680	0.9485	−0.0035 −0.1770 to 0.1701	0.9682	0.1676 −0.0061 to 0.3315	0.0586	0.1107 −0.0641 to 0.2789	0.2137

HOMA-IR INS: the homeostasis model assessment-estimated insulin resistance, insulin derived index; HOMA-IR CP1: the homeostasis model assessment-estimated insulin resistance, C-peptide 1 derived index; HOMA-IR CP2: the homeostasis model assessment-estimated insulin resistance, CP2 derived index; HOMA-BETA: the homeostasis model assessment of β-cell function; HOMA-BETA CP: the homeostasis model assessment of β-cell function, CP derived index; r: correlation coefficient; CI (95%): confidence interval of 95%; BMI: body mass index; HDL-cholesterol: high-density lipoprotein cholesterol; SBP: systolic blood pressure; DBP: diastolic blood pressure. Correlations were calculated with the Person test.

**Table 4:** Correlations of the MetS components with the metabolic indices: disposition index, QUICKI, 20/C-peptide*glucose, and C-peptide index.

	Disposition Index		QUICKI		20/C-Peptide*Glucose		C-Peptide Index	
	r CI (95%)	*p*-Value	r CI (95%)	*p*-Value	r CI (95%)	*p*-Value	r CI (95%)	*p*-Value
BMI								
	0.0131 −0.1608 to 0.1863	0.8829	−0.4695 −0.5946 to −0.3222	<0.0001	−0.0999 −0.2689 to 0.0749	0.2616	0.2200 0.0482 to 0.3791	0.0126
Waist circumference (cm)								
Females	−0.0096 −0.2475 to 0.2294	0.9378	−0.2761 −0.4828 to −0.0402	0.0227	−0.0363 −0.2725 to 0.2039	0.7685	0.0716 −0.1697 to 0.3049	0.5615
Males	0.0158 −0.2391 to 0.2687	0.9044	−0.5877 −0.7324 to −0.3922	<0.0001	−0.0135 −0.2666 to 0.2413	0.9182	0.2072 −0.0494 to 0.4381	0.1122
Glucose (mg/dL)								
	−0.8432 −0.8870 to −0.7845	<0.0001	−0.3603 −0.5025 to −0.1992	<0.0001	−0.1502 −0.3155 to 0.0240	0.0906	0.0974 −0.0774 to 0.2665	0.2738
HDL-cholesterol (mg/dL)								
Females	0.0439 −0.1966 to 0.2795	0.7217	0.2478 0.0099 to 0.4592	0.0416	0.1007 −0.1412 to 0.3312	0.4140	−0.1319 0.3591 to 0.1100	0.2836
Males	0.0452 −0.2959 to 0.2111	0.7312	0.3251 0.0775 to 0.5349	0.0113	−0.0535 −0.3034 to 0.2032	0.6847	−0.1715 −0.4077 to 0.0862	0.1901
Triglyceride (mg/dL)								
	−0.0506 −0.2223 to 0.1240	0.5700	−0.3615 −0.5035 to −0.2005	<0.0001	−0.0684 −0.2392 to 0.1064	0.4428	0.1038 −0.0710 to 0.2725	0.2435
Blood pressure (mmHg)								
SBP	0.1248 −0.0498 to 0.2921	0.1603	−0.0953 −0.2645 to 0.0795	0.2843	−0.0230 −0.1958 to 0.1511	0.7963	0.0918 −0.0830 to 0.2612	0.3027
DBP	0.1868 0.0136 to 0.3490	0.0348	−0.0529 −0.2245 to 0.1217	0.5526	−0.0478 −0.2196 to 0.1268	0.5919	0.0272 −0.1470 to 0.1999	0.7602

QUICKI: the quantitative insulin sensitivity check index; r: correlation coefficient; CI (95%): confidence interval of 95%; BMI: body mass index; HDL-cholesterol: high-density lipoprotein cholesterol; SBP: systolic blood pressure; DBP: diastolic blood pressure. Correlations were calculated with the Person test.

**Table 5:** Cut-off values for HOMA-IR INS, HOMA-BETA, and QUICKI to identify MetS.

Metabolic Index	Sex	Cut-Off Value	Sensitivity	Specificity	False Positive Rate (1—Specificity)
HOMA-IR INS	All	1.165	73.3%	43.9%	56.1%
		1.835	70.0%	67.3%	32.7%
		1.855	70.0%	68.4%	31.6%
	Females	0.725	77.8%	32.2%	67.8%
		1.690	66.7%	69.5%	30.5%
		1.805	66.7%	71.2%	28.8%
	Males	1.135	76.2%	33.3%	66.7%
		2.065	71.4%	69.2%	30.8%
		2.115	71.4%	71.8%	28.2%
HOMA-BETA	All	72.380	80.0%	46.9%	53.1%
		82.250	76.7%	54.1%	45.9%
		85.150	73.3%	54.1%	45.9%
	Females	49.240	77.8%	30.5%	69.5%
		69.770	66.7%	47.5%	52.5%
		71.305	66.7%	49.2%	50.8%
	Males	81.800	85.7%	46.2%	53.8%
		100.525	81.0%	56.4%	43.6%
		106.370	81.0%	59.0%	41.0%
QUICKI	All	0.385	80.0%	38.8%	61.2%
		0.375	73.3%	42.9%	57.1%
		0.355	70.0%	63.3%	36.7%
	Females	0.375	66.7%	49.2%	50.8%
		0.365	66.7%	57.6%	42.4%
		0.355	66.7%	67.8%	32.2%
	Males	0.385	85.7%	30.8%	69.2%
		0.375	76.2%	33.3%	66.7%
		0.345	71.4%	64.1%	35.9%

HOMA-IR INS: the homeostasis model assessment-estimated insulin resistance, insulin version; HOMA-BETA: the homeostasis model assessment of β-cell function; QUICKI: the quantitative insulin sensitivity check index.

**Table 6:** The correlations between hsCRP and the metabolic indices.

Index	Sex	r	CI (95%)	*p*-Value
HOMA-IR INS	All	0.1889	0.0158 to 0.3509	0.0328
	Females	0.0123	−0.2268 to 0.2501	0.9204
	Males	0.4405	0.2100 to 0.6246	0.0004
HOMA-IR CP1	All	0.2142	0.0422 to 0.3739	0.0152
	Females	0.2240	−0.0153 to 0.4390	0.0664
	Males	0.3101	0.0609 to 0.5229	0.0159
HOMA-IR CP2	All	0.2219	0.0502 to 0.3808	0.0118
	Females	0.2479	0.0100 to 0.4593	0.0415
	Males	0.3045	0.0547 to 0.5184	0.0180
HOMA-BETA	All	0.1808	0.0074 to 0.3436	0.0411
	Females	−0.0229	−0.2600 to 0.2167	0.8525
	Males	0.5018	0.2840 to 0.6704	<0.0001
HOMA-BETA CP	All	0.2102	0.0380 to 0.3703	0.0172
	Females	0.1219	−0.1201 to 0.3502	0.3220
	Males	0.3982	0.1604 to 0.5923	0.0016
Disposition index	All	0.0679	−0.1068 to 0.2387	0.4457
	Females	−0.0401	−0.2760 to 0.2002	0.7450
	Males	0.2486	−0.0057 to 0.4728	0.0554
QUICKI	All	−0.1189	−0.2865 to 0.0558	0.1814
	Females	−0.0200	−0.2573 to 0.2195	0.8709
	Males	−0.3297	−0.5386 to −0.0826	0.0101
20/C-peptide*glucose	All	−0.1735	−0.3370 to 0.0000	0.0501
	Females	−0.1892	−0.4092 to 0.0516	0.1223
	Males	−0.1616	−0.3992 to 0.0963	0.2174
C-peptide index	All	0.2293	0.0579 to 0.3874	0.0092
	Females	0.2190	−0.0205 to 0.4348	0.0728
	Males	0.3291	0.0819 to 0.5381	0.0102

r: correlation coefficient; CI (95%): confidence interval of 95%; HOMA-IR INS: the homeostasis model assessment-estimated insulin resistance, insulin derived index; HOMA-IR CP1: the homeostasis model assessment-estimated insulin resistance, C-peptide 1 derived index; HOMA-IR CP2: the homeostasis model assessment-estimated insulin resistance, C-peptide 2 derived index; HOMA-BETA: the homeostasis model assessment of β-cell function; HOMA-BETA CP: the homeostasis model assessment of β-cell function, CP derived index; QUICKI: the quantitative insulin sensitivity check index. The Pearson test was used to assess all the correlations.

## Data Availability

Data are contained within the article.

## Abbreviations

AT	Adipose tissue
BMI	Body mass index
CP	C-peptide
CPI	C-peptide index
CVD	Cardiovascular diseases
DI	Disposition index
HOMA-BETA	The homeostasis model assessment of β-cell function
HOMA-IR	The homeostasis model assessment-estimated insulin resistance
hsCRP	High-sensitivity C-reactive protein
INS	Insulin
WC	Waist circumference
MetS	Metabolic syndrome
T2DM	Type 2 diabetes mellitus
QUICKI	The quantitative insulin sensitivity check index
